# Supplementary material for: COZOID: contact zone identifier for visual analysis of protein-protein interactions
Source: BMC Bioinformatics. 2018 Apr 6;19:125. doi: 10.1186/s12859-018-2113-6 (PMC5889581; doi:10.1186/s12859-018-2113-6)
Supplement: Supplementary file 2 — Software build. Executable binary file of the software tool. (ZIP 118,784 kb) [file 12859_2018_2113_MOESM2_ESM.zip › cozoid/java/docs/beansbinding-1.2.1-doc.zip!/org/jdesktop/el/Expression.html]

Expression (Beans Binding)


---


|  |  |  |
| --- | --- | --- |
| **Overview** \| **Package** \| **Class** \| **Use** \| **Tree** \| **Deprecated** \| **Index** \| **Help** | |  |
| **PREV CLASS**   **NEXT CLASS** | **FRAMES**    **NO FRAMES**     **All Classes** |  |
| SUMMARY: NESTED \| FIELD \| CONSTR \| METHOD | DETAIL: FIELD \| CONSTR \| METHOD |  |


---


## org.jdesktop.el Class Expression

```
java.lang.Object
  org.jdesktop.el.Expression
```

**All Implemented Interfaces:**: java.io.Serializable

**Direct Known Subclasses:**: MethodExpression, ValueExpression

---

``` public abstract class Expression extends java.lang.Object implements java.io.Serializable ```

Base class for the expression subclasses `ValueExpression` and
`MethodExpression`, implementing characterstics common to both.

All expressions must implement the `equals()` and
`hashCode()` methods so that two expressions can be compared
for equality. They are redefined abstract in this class to force their
implementation in subclasses.

All expressions must also be `Serializable` so that they
can be saved and restored.

`Expression`s are also designed to be immutable so
that only one instance needs to be created for any given expression
String / `FunctionMapper`. This allows a container to pre-create
expressions and not have to re-parse them each time they are evaluated.

**Since:**
:   JSP 2.1

**See Also:**: Serialized Form

---

| **Nested Class Summary** | |
| --- | --- |
| `static class` | `Expression.ResolvedProperty` |
| `static class` | `Expression.Result` |


**Constructor Summary**

`Expression()`


| **Method Summary** | |
| --- | --- |
| `abstract  boolean` | `equals(java.lang.Object obj)`             Determines whether the specified object is equal to this `Expression`. |
| `abstract  java.lang.String` | `getExpressionString()`             Returns the original String used to create this `Expression`, unmodified. |
| `abstract  int` | `hashCode()`             Returns the hash code for this `Expression`. |
| `abstract  boolean` | `isLiteralText()`             Returns whether this expression was created from only literal text. |

**Methods inherited from class java.lang.Object**

`clone, finalize, getClass, notify, notifyAll, toString, wait, wait, wait`

**Constructor Detail**

### Expression

```
public Expression()
```


**Method Detail**

### getExpressionString

```
public abstract java.lang.String getExpressionString()
```

:   Returns the original String used to create this `Expression`,
    unmodified.

    This is used for debugging purposes but also for the purposes
    of comparison (e.g. to ensure the expression in a configuration
    file has not changed).

    This method does not provide sufficient information to
    re-create an expression. Two different expressions can have exactly
    the same expression string but different function mappings.
    Serialization should be used to save and restore the state of an
    `Expression`.

    :   **Returns:**: The original expression String.

---


### equals

```
public abstract boolean equals(java.lang.Object obj)
```

:   Determines whether the specified object is equal to this
    `Expression`.

    The result is `true` if and only if the argument is
    not `null`, is an `Expression` object that
    is the of the same type (`ValueExpression` or
    `MethodExpression`), and has an identical parsed
    representation.

    Note that two expressions can be equal if their expression
    Strings are different. For example, `${fn1:foo()}`
    and `${fn2:foo()}` are equal if their corresponding
    `FunctionMapper`s mapped `fn1:foo` and
    `fn2:foo` to the same method.

    :   **Overrides:**: `equals` in class `java.lang.Object`
    :   **Parameters:**: `obj` - the `Object` to test for equality. **Returns:**: `true` if `obj` equals this `Expression`; `false` otherwise. **See Also:**: `Hashtable`, `Object.equals(java.lang.Object)`

---


### hashCode

```
public abstract int hashCode()
```

:   Returns the hash code for this `Expression`.

    See the note in the `equals(java.lang.Object)` method on how two expressions
    can be equal if their expression Strings are different. Recall that
    if two objects are equal according to the `equals(Object)`
    method, then calling the `hashCode` method on each of the
    two objects must produce the same integer result. Implementations must
    take special note and implement `hashCode` correctly.

    :   **Overrides:**: `hashCode` in class `java.lang.Object`
    :   **Returns:**: The hash code for this `Expression`. **See Also:**: `equals(java.lang.Object)`, `Hashtable`, `Object.hashCode()`

---


### isLiteralText

```
public abstract boolean isLiteralText()
```

:   Returns whether this expression was created from only literal text.

    This method must return `true` if and only if the
    expression string this expression was created from contained no
    unescaped EL delimeters (`${...}` or
    `#{...}`).

    :   **Returns:**: `true` if this expression was created from only literal text; `false` otherwise.


---


|  |  |  |
| --- | --- | --- |
| **Overview** \| **Package** \| **Class** \| **Use** \| **Tree** \| **Deprecated** \| **Index** \| **Help** | |  |
| **PREV CLASS**   **NEXT CLASS** | **FRAMES**    **NO FRAMES**     **All Classes** |  |
| SUMMARY: NESTED \| FIELD \| CONSTR \| METHOD | DETAIL: FIELD \| CONSTR \| METHOD |  |


---
